# 液体活检在非小细胞肺癌中的临床应用进展

**DOI:** 10.3779/j.issn.1009-3419.2021.102.33

**Published:** 2021-10-20

**Authors:** 敏 张, 骏 陈

**Affiliations:** 116000 大连，大连医科大学附属第二医院肿瘤科 Department of Oncology, The Second Hospital of Dalian Medical University, Dalian 116000, China

**Keywords:** 肺肿瘤, 液体活检, 循环肿瘤DNA, 循环肿瘤细胞, 外泌体, Lung neoplasms, Liquid biopsy, Circulating tumor DNA, Circulating tumor cells, Exosomes

## Abstract

肺癌是我国发病率最高的癌症，也是癌症患者死亡的首要病因。其中，约85%是非小细胞肺癌（non-small cell lung cancer, NSCLC）。肺癌患者的诊断方法和治疗措施一直是重中之重。近年来，随着分子生物学检测技术的快速进步以及精准治疗和个体化治疗的发展，液体活检因其具有传统侵入性活检所不具备的优点而成为关注的焦点，如安全、便捷、重复性、低创伤性等。液体活检作为一种新兴技术，其检测对象主要包括外周血中的循环肿瘤DNA（circulating tumor DNA, ctDNA）、循环肿瘤细胞（circulating tumor cells, CTCs）、外泌体等。本文将对ctDNA、CTCs和外泌体的检测及其在NSCLC患者中的临床应用作一简要综述。

## Abstract (en)

Lung cancer, with the highest incidence in China, is the leading cause of death in cancer patients. Of these, about 85% are patients with non-small cell lung cancer (NSCLC). Therefore, the diagnosis and treatment of patients with lung cancer have always been a top priority nowadays. Fluid biopsy has many advantages, such as safety, convenience, repeatability, low trauma and so on, which are not available in traditional invasive biopsy. In recent years, with the rapid progress of molecular biological detection technology, fluid biopsy, as a new technology, has become the focus of attention. What's more, it contributes to the development of precision treatment and individualized treatment of lung cancer. Liquid biopsy mainly detects circulating tumor DNA (ctDNA), circulating tumor cells (CTCs) and exosomes in peripheral blood. We will make an introduce to the detection and clinical applications of ctDNA, CTCs and exocrine in this article, in order that it can provide insights into future clinical treatment for NSCLC.

肺癌是最常见的恶性肿瘤之一，世界卫生组织国际癌症研究机构发布，2020年全球肺癌发病率为11.4%，死亡率为18.0%，已成为恶性肿瘤致死的首要原因^[1]^。其中非小细胞肺癌（non-small cell lung cancer, NSCLC）是肺癌的一种最常见的类型，约占肺癌总数的85%^[2]^。肺癌患者主要是通过组织病理活检进行明确诊断，然而组织活检具有较大创伤性且操作起来相对复杂，对于一些术后或转移性肺癌患者来说，肿瘤体积太小或肿瘤位置不适合进行组织活检。另外组织活检只能反映肿瘤在某一时间点的局部状态^[3]^，这样可能会错过某些肿瘤突变信息。液体活检是指从体液中获得来源于组织的生物标志物，并通过对所得的生物标志物进行分析得到其来源组织的相关信息^[4]^，在肿瘤领域具有巨大潜力和广泛临床应用前景。本文将从液体活检在NSCLC的筛查、早期诊断、监测病情变化、预测预后、探索耐药机制以及肺癌疫苗的研制等方面进行阐述。

## 1 液体活检主要检测对象

### 1.1 循环肿瘤DNA（circulating tumor DNA, ctDNA）

ctDNA是指人体血液循环系统中不断流动的携带一定特征，包括突变、缺少、插入、重排、拷贝数异常和甲基化等来自肿瘤基因组的DNA片段^[5]^。ctDNA的主要来源包括：坏死的肿瘤细胞、凋亡的肿瘤细胞、循环的肿瘤细胞、肿瘤细胞分泌的外泌体。当癌细胞坏死或凋亡时释放出漂浮在血液中的基因组片段即ctDNA。在晚期癌症患者中，肿瘤可能是血液中大部分循环游离DNA（cell-free DNA, cfDNA）的来源^[6]^。ctDNA是一种敏感性和特异性较强的生物标志物，广泛应用于多种不同类型肿瘤的临床研究^[7]^。但由于ctDNA所占比例较小，从正常cfDNA中难以区分ctDNA，对样本中突变片段数量进行准确定量存在困难，因此需要灵敏度和特异性较高的检测方法^[8]^。过去DNA测序方法主要是第一代DNA测序方法和焦磷酸测序法。近年来，随着二代测序（next-generation sequencing, NGS）技术和数字PCR（digital PCR, dPCR）技术的发展改变了检测ctDNA的格局。目前，对于已知突变的ctDNA主要检测技术有dPCR技术，基于小珠、乳浊液、扩增和磁性（bead, emulsion, amplification and magnetic, BEAMing）的技术以及突变阻滞扩增系统PCR（amplification refractory mutation system PCR, ARMS-PCR）技术。而对于突变位点未知的主要使用标记扩增深度测序（tagged-amplicon deepsequencing, TAm-Seq）技术和癌症个体化深度测序分析（cancer personalized profiling by deep sequencing, CAPP-Seq）技术。

### 1.2 循环肿瘤细胞（circulating tumor cells, CTCs）

CTCs是从原发性或转移性肿瘤组织流入外周血液的罕见癌细胞。这些CTCs的表型和遗传特征可以为指导癌症分期和治疗提供重要信息，因此进一步研究它们的特征和性质是一个相当有价值的领域，也为液体活检提供细胞来源^[9, 10]^。在外周血中，约每10^6^个-10^7^个白细胞中可能仅有1个CTCs^[11]^，因此CTCs富集分离是首要问题。CTCs检测基本过程是先对其进行分离富集，再进行分析。富集和分析的方法基于CTCs的物理和生物学特性，例如大小、变形性、密度、极性和电荷、上皮细胞黏附分子、细胞角蛋白和表达的肿瘤相关标志物^[12]^。CTCs富集的方法主要有：密度依赖性细胞分离、基于细胞大小的分离、免疫磁珠阴性富集、基于磁泳移动性的分离、免疫磁珠阳性富集和通过微流体装置富集。对CTCs进行分析的方法有：逆转录PCR技术、荧光原位杂交技术和荧光辅助细胞分选技术。另外可同时富集和分析CTCs的方法有：光纤阵列扫描技术、CellSearch、基于形态学富集的膜过滤技术（isolating by size of epithelial tumor cells, ISET）、AdnaTest和EPISPOT等。目前还有一些新兴技术如利用声波定向微流体将肿瘤细胞与白细胞分离，有助于我们快速准确地从全血中分离和分析CTCs^[13]^。

### 1.3 外泌体

1983年，外泌体最先在绵羊的网织红细胞中被发现^[14]^。外泌体是一种细胞源性囊泡，直径为30 nm-100 nm，它可以从血浆、唾液、尿液、乳汁、胸水、脑脊液和精液等多种体液中检测到其内含有的蛋白质（如细胞因子、生长因子）和miRNA等生物活性物质，通过对肿瘤外泌体的检测分析可以得到肿瘤细胞的相关信息^[15, 16]^。外泌体提取的方法有：差速超速离心法、PEG沉淀法、声波分离法、密度梯度离心法、过滤离心法、吸附法和免疫磁珠法等。外泌体的鉴定方法包括：电子显微镜、免疫金标记技术、实时荧光定量聚合酶链式反应、流式细胞仪、纳米颗粒追踪分析技术和测序等。

## 2 液体活检可用于NSCLC患者的筛查及早期诊断

NSCLC发病率和死亡率较高，且大部分患者在确诊时分期较晚，因此筛查和早期诊断至关重要^[1, 2]^。早期诊断和筛查常采用影像学方法，尽管低剂量螺旋计算机断层扫描（computed tomography, CT）筛查灵敏度高，但其特异性相对较低，导致其过度诊断概率达18.5%^[17]^。现在液体活检已被认为可以代替肿瘤组织，用于肿瘤相关遗传和表观遗传改变的非侵入性检测，并且已进入临床应用^[18-21]^。Ilie等^[18]^进行了一项前瞻性研究，利用ISET技术检测慢性阻塞性肺气肿患者血液中的CTCs，并跟踪随访入组患者1年-4年。结果发现CTCs阳性的患者中有5例患者通过CT扫描确定有肺结节，随后的手术切除和病理检查确诊为早期肺癌；CTCs阴性的对照组在同一时期未发现肺癌。同样有研究者招募了614例慢性阻塞性肺气肿的患者，采集基线血液样本，同样利用ISET技术来检测CTCs，然后进行跟踪随访。研究^[19]^结果显示，基线CTCs检测对肺癌检测的敏感性为26.3%，提示CTCs可以用作肺癌筛查的潜在生物标志物。

另外Jin等^[20]^通过分离血浆的肿瘤来源的外泌体，对60例参与者的miRNA-seq数据进行分析从而得到NSCLC早期诊断价值的评估，60例参与者中有43例是NSCLC患者，用于NSCLC诊断的miRNA在区分NSCLC患者和非NSCLC患者中的灵敏度为80.25%，特异性为92.31%。其中用于腺癌诊断的两种miRNA（miR-181-5p, miR-361-5p）在区分腺癌患者灵敏度为80.65%，特异性为91.67%。用于鳞癌诊断的两种miRNA（miR-320b, miR-10b-5p）在区分鳞癌患者灵敏度为83.33%，特异性为90.32%。另外有研究^[21]^发现在肺癌的发生发展过程中，环状RNA circSATB2在肺癌患者的血清外泌体中高表达，对临床检测具有高度敏感性和特异性。circSATB2可以被外泌体转移，促进NSCLC细胞的增殖、迁移和侵袭，并在正常人支气管上皮细胞中诱导其异常增殖，与肺癌转移有关。可见通过对外泌体的分析有助于NSCLC患者的早期筛选和诊断，并且有助于鉴别腺癌和鳞癌患者。

## 3 液体活检可用于监测NSCLC患者病情变化

NSCLC患者可以发生复发、进展和转移，有创的组织活检只能反映肿瘤某一时间点的局部状态，并不能完全代表患者的全部病情，且穿刺可能会引起种植转移。临床上主要是通过影像学检查对肿瘤患者的治疗效果进行评价。液体活检可能允许患者在治疗过程中实时取样来评估病情，可实时监测肿瘤在分子水平的动态变化，及时掌握肿瘤状态和病情变化，提高疗效评价的准确度，从而更好地调整治疗方案^[22]^。Aggarwal等^[23]^的一项前瞻性研究纳入了323例转移性NSCLC患者，用NGS检测患者血浆和组织的基因突变。其中94例患者只进行血浆检测，31例患者检测到可接受靶向治疗的基因突变，因此无需进行侵入性组织活检。有128例患者同时进行了血浆和组织的NGS检测，8例在血浆中检测到可靶向治疗的基因突变，而其组织检测结果为野生型，可见血浆检测提高了检测的突变率。对于无法进行组织活检的101例患者，27例患者检测到可治疗靶向突变。在基于血液检测结果接受靶向治疗的42例患者中有36例实现了完全缓解、部分缓解和病情稳定。这项临床研究是衡量基于血浆的基因分型对NSCLC靶向治疗的影响的研究之一，提示液体活检提高了基因检测的检出率，可以作为一种重要手段以期提高靶向治疗效果。

免疫检查点抑制剂在晚期NSCLC患者中显示出卓越的疗效。免疫治疗的选择是基于通过免疫组化检测肿瘤程序性死亡受体-配体1（programmed cell death-ligand 1, PD-L1）表达，然而用于预测免疫治疗疗效的潜在生物标志物仍不确定。一项研究^[24]^测定血浆来源外泌体中PD-L1 mRNA水平与NSCLC患者免疫治疗效果之间的关系。在基线和治疗2个月后从患者血浆中提取外泌体，通过dPCR检测PD-L1 mRNA表达水平，并对患者病情重新评估。结果显示外泌体中PD-L1 mRNA水平根据肿瘤对药物反应而发生变化，疾病进展的患者PD-L1 mRNA增加，而病情部分缓解或完全缓解的患者PD-L1 mRNA减少。接受免疫治疗的NSCLC患者中外源性PD-L1表达发生了变化，并且可能与患者治疗效果相关，证明了检测血浆外泌体PD-L1的可行性及其与免疫治疗效果的关系。免疫治疗与抗血管生成治疗的结合在多线治疗中也显示出很好的疗效，有研究^[25]^纳入了22例在化疗、放疗、靶向治疗、手术和其他治疗方法中均失败的晚期NSCLC患者，这些患者接受卡瑞利珠单抗免疫治疗联合阿帕替尼抗血管生成治疗。研究发现血液肿瘤突变负荷无法区分从这种联合治疗受益的患者，而ctDNA中变异等位基因则是治疗效果的敏感指标，可用于监测肿瘤的缓解或进展。另外，有研究^[26]^发现ctDNA测序可以在CT扫描诊断肿瘤复发前70 d检测出NSCLC复发，并且可以识别原发性肿瘤的不同克隆以及转移性肿瘤的分子变化。无论是靶向治疗、免疫治疗或联合治疗，液体活检均发挥一定的价值。液体活检的临床应用可以减少患者再次组织活检的有创损伤，可以实时监测病情变化，甚至可以提前发现患者的病情进展。

## 4 液体活检可用于预测NSCLC患者预后

早期肺癌患者术后复发是难以攻克的问题，有研究者从24例接受手术的I期-IIIa期肺癌患者收集术前血浆样本，使用微滴式数字PCR技术分析血浆样品的ctDNA，然后跟踪随访目标患者至少2年。在术前cfDNA中可检测到体细胞改变的7例患者中有2例发生了早期复发（2/7, 29%）。相比之下，手术时未检测到ctDNA的12例患者中有2例在随访期内出现肿瘤复发（2/12, 17%）。术前ctDNA分析正确预测了一半的复发患者（敏感性为50%，特异性为67%）^[27]^。目前有大量研究显示CTCs对各种类型实体瘤患者中的预后具有指导意义，Yoneda等^[28]^对94例手术完全切除的原发性肺癌患者进行长期随访并检测CTCs。在16例患者中检测到CTCs，CTCs阳性与无复发生存率呈负相关，CTCs阳性也与较差的总生存率相关，CTCs阳性与术后肺癌患者的无复发生存率和总体生存率低有关。可见液体活检在早期肺癌患者预后方面表现出独特的优势。

液体活检在预测晚期NSCLC患者预后方面也不可或缺。在接受化疗的晚期NSCLC患者中，有研究发现CTCs数量可以提前预判出难以通过影像学检测到的细微转移和复发性肿瘤，与NSCLC患者预后密切相关，CTCs数量≤5个的患者的无进展生存期（progression-free survival, PFS）为11.3个月，而CTCs数量 > 5个的患者PFS仅为7.2个月^[29]^。另外，Coco等^[30]^为了研究cfDNA和CTCs在接受一线化疗的NSCLC患者中的预后作用，纳入了73例晚期NSCLC患者。在基线和2个化疗周期后分析cfDNA和CTCs，通过qPCR进行血浆cfDNA定量。结果显示基线cfDNA高于中位值的患者总生存期（overall survival, OS）明显更差，死亡风险增加1倍。同时观察到基线CTCs中位数与OS之间呈反比关系。此外，在病情稳定的患者中，基线的cfDNA和CTCs能够鉴别出高风险NSCLC患者，且在总体人群中cfDNA是比CTCs更可靠的生物标志物。在接受免疫治疗的NSCLC患者中也同样的发现CTCs计数以及代谢参数是PFS和OS的预后因素^[31]^。Nicolazzo等^[32]^招募了24例使用Nivolumab进行治疗的IV期NSCLC患者，发现CTCs的数量和其表面上的PD-L1的表达与患者的不良预后相关。另有数据^[33]^显示ctDNA在早期治疗时变化可能是免疫治疗获益的有用预测指标。在靶向治疗方面，有研究者把154例接受吉非替尼治疗的NSCLC患者作为观察组，52名健康人作为对照组。研究结果发现NSCLC患者治疗前血浆cfDNA水平明显高于对照组；在观察组的NSCLC患者中，随着病情严重程度的增加，cfDNA水平也逐渐升高；cfDNA低表达组患者的生存期明显高于高表达组。可以看出cfDNA浓度是影响NSCLC预后的独立因素，血浆cfDNA是NSCLC患者的潜在预后指标^[34]^。可见液体活检可以应用于接受手术、化疗、免疫治疗和靶向治疗的NSCLC患者，并且可以通过检测cfDNA或CTCs等来进一步预测患者的预后。

## 5 液体活检可用于探索NSCLC耐药机制

肿瘤患者耐药一直是难以突破的难题，尤其是靶向治疗。靶向治疗在具有特定突变基因的肺癌患者中有着显著的临床效果，但在治疗过程中不可避免地发生耐药，肿瘤异质性导致靶向治疗期间多种耐药机制的发展。因此，在NSCLC患者治疗过程中基因突变动态纵向检测，对于指导疾病进展或耐药发生后的治疗变得越来越重要。Tsui等^[35]^研究纳入了接受吉非替尼和羟氯喹治疗的50例表皮生长因子受体（epidermal growth factor receptor, *EGFR*）基因突变的NSCLC患者，纵向分析其中45例NSCLC患者的ctDNA动态变化。一组患者在疾病进展前后都保留*EGFR*敏感突变，其血浆样本中检测到T790M突变，表明至少有一些对TKI产生耐药性的患者是通过T790M突变导致疾病发生进展的。在另一组患者中，进展前后都在血浆中检测到*EGFR*突变，但没有发生T790M突变，表明存在其他机制产生耐药性。在余下的患者中，疾病进展时*EGFR*突变消失，也没有在血浆中检测到*EGFR*-T790M耐药突变。可见对NSCLC患者血浆ctDNA纵向分析揭示了耐药机制的异质性，可以帮助确定耐药机制，指导临床治疗。同样CTCs作为识别基因突变的液体活检的出现也为肺癌患者治疗和监测提供了新的机会。在间变性淋巴瘤激酶（anaplastic lymphoma kinase, *ALK*）阳性肺腺癌的患者的CTCs中检测到棘皮动物微管相关类蛋白4（echinoderm microtubule-associated protein-like 4, *EML4*）-*ALK*基因重排。使用克唑替尼进行治疗过程中，患者病情进展对ALK-TKI产生耐药，此时从外周血CTCs中监测到耐药性基因突变，即*ALK*基因上的L1196M^[36]^。研究表明患者外周血CTCs的检测可以解释患者发生耐药的机制并且为下一步治疗的调整提供依据。

有研究者从T790M突变型NSCLC细胞株H1975和敏感细胞株PC9的条件培养基中分离出外泌体，并分析外泌体miRNA表达谱。发现H1975分泌的外泌体可通过激活PI3K/AKT信号通路体外使PC9获得吉非替尼耐药性。miR-3648和miR-522-3p是表达最差的两个miRNA。功能研究^[37]^表明，miR-522-3p的上调可诱导PC9细胞对吉非替尼的耐药性。研究的结果揭示了NSCLC中EGFR-TKIs获得性耐药的重要机制。NSCLC患者在治疗期间会不可避免地出现特异性耐药机制，使得通过再次进行组织活检来获得处于进展中的肿瘤组织变得至关重要。鉴于诊断样本的缺乏和进行侵入性手术困难等问题，肿瘤组织并非总是可以获得用于检测耐药，因此液体活检的应用成为了克服这些困难的有效方法。

## 6 液体活检可用于指导肺癌疫苗的研制

液体活检除了可以用于NSCLC患者的筛查、早期诊断、监测病情变化、判断预后以及探索耐药机制外，还可以指导肺癌疫苗的研制。细胞可以将细胞外囊泡释放到周围环境中以相互沟通，外泌体是细胞外囊泡的亚组，源自内吞途径，它们含有可在细胞间转移的RNA分子、蛋白质和脂质。已经有研究^[38]^证明外泌体具有调节免疫应答的能力，因此外泌体被认为是有益的癌症和感染的无细胞疫苗。研究表明肿瘤细胞起源的外泌体、树突状细胞起源的外泌体以及腹水细胞来源的外泌体有可能为肺癌的疫苗研制带来曙光。肿瘤来源的外泌体可以通过触发更强的树突状细胞介导的免疫反应和减少肿瘤微环境中的调节性T细胞来改善疫苗介导的免疫治疗^[39]^。树突状细胞起源的外泌体可以通过增强抗肿瘤T细胞反应、抑制癌细胞增殖以及根除肿瘤细胞从而增强肺癌患者免疫系统的反应^[40]^。此外，树突状细胞起源的外泌体具有比树突状细胞更长的体内半衰期，不受免疫调节剂的影响，并且没有基于细胞的疫苗的风险。因此，可以进行临床试验以测试由携带肿瘤抗原的树突状细胞起源的外泌体组成的疫苗的可行性，安全性和功效^[41]^。

## 7 小结与展望

与传统组织活检相比，液体活检技术具有便捷、无创性、可重复性等优势。诸多研究表明液体活检作为精准医疗的新技术在肿瘤早期诊断、进展与转移、异质性与耐药性以及预后评估等领域具有独特的技术优势和广泛的临床应用价值。通过检测ctDNA、CTCs和外泌体等可以得到组织活检无法实时提供的信息，并为实现肺癌患者的个体化诊疗提供有利的循证医学依据，使患者受益。但在临床中，不能单一的将某项指标作为评价标准，应该将液体活检数据与组织活检、影像学结果、肿瘤标志物等临床指标相结合，综合性地实时监测指导NSCLC患者的临床治疗，为患者的实际情况提供更加精确的信息。

虽然cfDNA可以作为靶向治疗患者的循环标志物，但CTCs、miRNA、外泌体和肿瘤血小板仍处于临床前阶段。尽管它们在筛选程序中作为预后、预测性生物标志物显示出喜人的结果，但是仍面临一些挑战。一方面，ctDNA、CTCs和外泌体等在血液中含量较低，对检测技术的灵敏度要求较高；另一方面，较高的检测成本可能会影响液体活检的广泛应用，必须进行大型的前瞻性临床试验以提供其临床效用的证据。相信随着检测手段的不断进步、检测灵敏度和稳定性提高，检测技术困难终将被攻克。届时液体活检技术能够帮助在诊断和治疗方面实现突破，同时能够指导临床方案的制定，实现真正的个体化精准医疗。
